# TRPA1 Role in Inflammatory Disorders: What Is Known So Far?

**DOI:** 10.3390/ijms23094529

**Published:** 2022-04-20

**Authors:** Lorenzo Landini, Daniel Souza Monteiro de Araujo, Mustafa Titiz, Pierangelo Geppetti, Romina Nassini, Francesco De Logu

**Affiliations:** Department of Health Sciences, Clinical Pharmacology and Oncology Section, University of Florence, 50139 Florence, Italy; l.landini@unifi.it (L.L.); daniel.souzamonteirodearaujo@unifi.it (D.S.M.d.A.); mustafa.titiz@unifi.it (M.T.); geppetti@unifi.it (P.G.); francesco.delogu@unifi.it (F.D.L.)

**Keywords:** TRPA1, inflammatory cells, immune cells, inflammatory diseases

## Abstract

The transient receptor potential ankyrin 1 (TRPA1), a member of the TRP superfamily of channels, is primarily localized in a subpopulation of primary sensory neurons of the trigeminal, vagal, and dorsal root ganglia, where its activation mediates neurogenic inflammatory responses. TRPA1 expression in resident tissue cells, inflammatory, and immune cells, through the indirect modulation of a large series of intracellular pathways, orchestrates a range of cellular processes, such as cytokine production, cell differentiation, and cytotoxicity. Therefore, the TRPA1 pathway has been proposed as a protective mechanism to detect and respond to harmful agents in various pathological conditions, including several inflammatory diseases. Specific attention has been paid to TRPA1 contribution to the transition of inflammation and immune responses from an early defensive response to a chronic pathological condition. In this view, TRPA1 antagonists may be regarded as beneficial tools for the treatment of inflammatory conditions.

## 1. Introduction

The transient receptor potential (TRP) family of channels includes non-selective cation channels that are represented by 28 different members grouped in 6 subfamilies, namely: canonical (TRPC), vanilloid (TRPV), melastatin (TRPM), mucolipin (TRPML), polycystin (TRPP), and ankyrin (TRPA). TRP channels are activated by highly heterogenous, exogenous, and endogenous stimuli, such as changes in temperature and pH, reactive oxygen species (ROS), osmotic stress, and bacterial toxins or chemical compounds [1]. Their activation is essential in several sensory transduction pathways [2], homeostatic functions [3], and physiological and pathophysiological processes [1,3]. Given their expression in sensory neurons, some TRP channels have been associated with the detection and signaling of painful stimuli. However, recent findings have shown TRP channel expression in a variety of immune and inflammatory cells, such as dendritic cells, macrophages, and T lymphocytes [4], and remarkable crosstalk between nerve fibers and immune/inflammatory cells in the regulation of inflammatory processes [5,6,7,8].

Among the different TRPs, TRPM2 is widely expressed in immune cells, such as neutrophils, where it modulates chemotaxis in different inflammatory conditions [9,10]. TRPM7 also contributes to neutrophil recruitment, as the receptor’s kinase domain is important for neutrophil chemotaxis [11]. TRPV4 is expressed in monocytes, neutrophils, and T lymphocytes [12], and it has recently been detected in bone marrow-derived macrophages [13]. TRPV4 has been proposed to mediate neutrophil adhesion and chemotaxis and increase ROS production under inflammatory conditions [14]. Furthermore, in macrophages, TRPV4 activation releases ROS and reactive nitrogen species (RNS), which are among the essential mediators of immune responses [15,16,17]. In a mouse model of sepsis, TRPV1 deletion resulted in impaired phagocytes defense mechanisms, thus implying that TRPV1 acts to protect against the inflammatory condition [18]. An increased expression of TRPV1 has been observed in bone marrow-derived macrophages after stimulation by oxidized low-density lipoprotein, followed by intracellular calcium (Ca^2+^) increase [19].

## 2. Transient Receptor Potential Ankyrin 1 (TRPA1)

**Figure 1:**
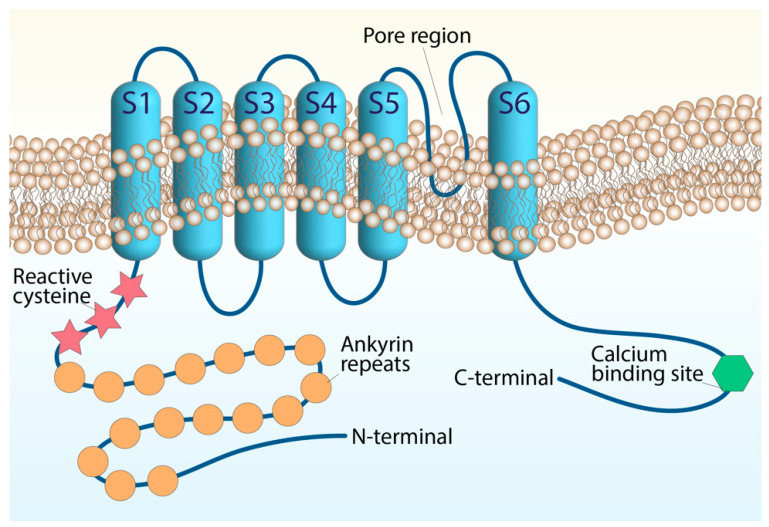
Structure of the TRPA1 channel. The TRPA1 architecture containing six transmembrane domains and intracellular N- and C-terminals. The transmembrane S5–S6 forming the central pore and selectivity filter. The reactive cysteine residues are within the N-terminal domain, along with the N-terminal ankyrin repeats, and the calcium-binding region is within the C-terminal.

The ankyrin-like protein with transmembrane domains (ANKTD), initially identified in lung fibroblasts [20], has been successively included in the TRP superfamily (renamed TRPA1, A standing for ankyrin) for its strong homology with several components of the superfamily. In humans, the *trpa1* gene consists of 27 exons and spans 55,701 base pairs of the human chromosome 8q13 [21,22]. Like all TRP channels, TRPA1 has six transmembrane domains (S1–S6) with a pore region between S5 and S6 and cytoplasmic N- and C-terminals associated with homo-tetramers. The peculiarity of TRPA1 is an unusually elongated (14–18) ankyrin repeat domain within the N-terminal, which connects with transmembrane proteins to the cytoskeleton and is involved in protein–protein interactions, as well as in channel trafficking to the plasma membrane (Figure 1).

N-terminal cysteine residues represent important functional sites of the channel. The cysteine residues allow channel activation by endogenous mediators, such as oxidative stress byproducts and exogenous electrophiles, through their oxidative modification [22,23,24,25,26,27,28]. However, TRPA1 possesses additional domains that appear to be crucial for its function. A putative EF-hand motif has been identified in the N-terminal region and represents the most common mechanism for many Ca^2+^-interacting proteins. Intracellular Ca^2+^ ions potentiate agonist-induced responses and directly activate the channel, probably through this mechanism [29,30], although its functional relevance is still being debated. TRPA1 channel activity undergoes modulation by negatively charged ligands, including phosphoinositides or inorganic polyphosphates [31], interacting with a yet-unidentified positively charged domain in the C-terminal region. Basic residues in the C-terminal, strongly involved in TRPA1 voltage and chemical sensitivity, may represent the possible interaction sites for negatively charged molecules generally considered to modulate TRPA1 [31]. TRPA1 subunits can assemble into hetero-tetrameric complexes with TRPV1 to adapt to the single-channel biophysical properties in native sensory neurons [1,32].

**Figure 2:**
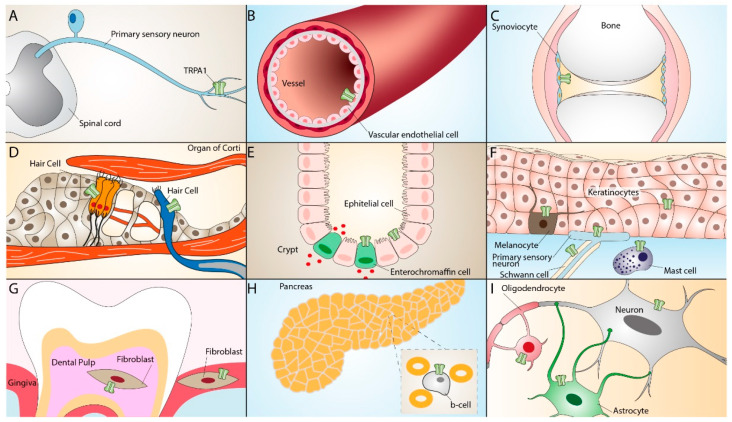
TRPA1 expression in neuronal and non-neuronal tissues. TRPA1 expression in primary sensory neurons (**A**), vascular endothelial cells (**B**), synoviocytes (**C**), inner ear hair cells (**D**), enterochromaffin cells, epithelial cells (**E**), keratinocytes, melanocytes, Schwann cells, mast cells (**F**), dental pulp and gingiva fibroblasts (**G**), pancreatic β-cells (**H**), oligodendrocytes, and astrocytes (**I**).

TRPA1 is widely distributed in neural (primary sensory neurons) [33] and non-neural tissues, including mouse inner ear and the organ of Corti [34], rat vascular endothelial cells [35], enterochromaffin cells [36], cells of the respiratory tract [37,38,39], human keratinocytes, melanocytes, synoviocytes, and gingival fibroblasts [40]. The TRPA1 receptor can also be found in epithelial cells, mast cells, and pancreatic β cells [41,42,43,44,45,46,47]. More recent studies have reported the presence of TRPA1 in glial cells, such as astrocytes [48], oligodendrocytes [49,50], and Schwann cells [51,52], where it contributes to different regulatory and proinflammatory pathways [53,54,55] (Figure 2).

Worthy of note, TRPA1 is activated by a wide variety of exogenous irritants that cause pain and inflammation [33]. The channel is also targeted by endogenous inflammatory agents, such as ROS, 4-hydroxynonenal (4-HNE) and 4-oxononenal (4-ONE) [27], and reactive nitrogen species (RNS) that can produce nitrated fatty acids, such as nitrooleic acid [56]. Prostaglandins are important fatty acid derivatives that are produced locally at sites of inflammation and tissue injury [57]. Some arachidonic acid derivatives, such as cyclopentenone prostaglandins, have been proposed to directly activate TRPA1 [58,59]. The prominent role of TRPA1 in inflammation is also underlined by its presence in a subset of peptidergic primary afferents. TRPA1 activation on these neurons promotes the release of the vasoactive and proinflammatory neuropeptides, substance P (SP), and calcitonin gene-related peptide (CGRP) [26] from central and peripheral terminals, thus promoting neurogenic inflammation [27,60]. CGRP release is considered a critical factor in the genesis of migraine pain [61,62]. TRPA1 ability to stimulate CGRP release from trigeminal terminals and the ensuing neurogenic inflammation in migraine has been proposed and discussed in previous papers [63,64] and will not be the object of the present review.

Given its expression and function in many different types of tissues and cells, TRPA1 operates as a sensor of cell stress, tissue injury, and exogenous noxious stimuli, and its activation leads to defensive responses. However, under circumstances of aberrant regulation, TRPA1 may exacerbate tissue inflammation and its consequences. Here, we summarize the recently discovered functional properties of TRPA1 in its beneficial/detrimental roles in several inflammatory disorders.

## 3. TRPA1 and Asthma and Chronic Obstructive Pulmonary Disease (COPD)

Asthma and chronic obstructive pulmonary disease (COPD) are common airway diseases characterized by airway obstruction hyperresponsiveness (AHR), persistent respiratory symptoms, and airflow limitation in association with airway inflammation [65]. Asthma is also one of the most common causes of chronic cough [66,67]. A hallmark of asthma and COPD is a generation/imbalance between oxidants and antioxidants (oxidative stress) [68,69,70,71], which may be generated from endogenous mechanisms (oxidative bursts from activated neutrophils and macrophages) or may derive from exogenous sources of oxidant species, including cigarette smoke, occupational and environmental pollutants, and other chemicals.

The pathophysiological role of TRPA1 in the respiratory tract seems primarily dependent on neurogenic inflammation [72,73]. Lungs are predominantly innervated by vagal sensory neurons activated by mechanical and chemical stimuli [74]. Noxious stimuli are detected by receptors expressed by nociceptors that sense environmental and internal signals to regulate bronchoconstriction, breathing patterns, vasodilatation, mucus production, and inflammation [75].

Various noxious chemicals and environmental/industrial irritants that activate TRPA1 function as triggers for airway inflammatory diseases and are known to worsen asthma attacks [76]. These chemicals include industrial pollutants (e.g., isocyanates, heavy metals, and oxidizing agents) and general anesthetics, which can cause neurogenic inflammation in local airways through a TRPA1-dependent mechanism [33,38,77]. Cigarette smoke contains many reactive molecules, such as crotonaldehyde, acrolein, acetaldehyde, and nicotine [78,79,80], expressed by airway sensory nerve terminals to release proinflammatory neuropeptides, cytokines, and chemokines. In rodents, these reactive molecules in cigarette smoke activate an early neurogenic inflammatory response, which is entirely mediated by TRPA1 [78].

Gram-negative bacterial infections are often accompanied by inflammation and somatic or visceral pain. These symptoms have been attributed to the sensitization of nociceptors by inflammatory mediators released by immune cells through activation of TRPA1-dependent mechanism by bacterial (lipopolysaccharides) LPS [81]. In addition, LPS can release neuropeptides by a TRPA1-dependent mechanism from nociceptive sensory neurons, thus inducing airway neurogenic inflammation [82].

TRPA1 has been critically proposed to contribute to airway inflammation in asthma. In an ovalbumin (OVA) mouse model of asthma, genetic ablation or pharmacologic inhibition of the TRPA1 channel attenuates the increase in several biochemical and functional markers in sensitized mice. Inflammatory cells were markedly reduced in TRPA1-deficient mice or after HC-030031 pretreatment [83]. A contribution of TRPA1 to airway inflammation has also been reported in a rat model of asthma. The immune cell infiltration observed in the rat lung after the OVA challenge was reduced in TRPA1 knockout rats in a manner similar to that observed in TRPA1 knockout mice [84], thus supporting the role of TRPA1 in inflammatory airway disease. There is also evidence that, in humans, TRPA1 polymorphisms correlate with reduced asthma control [85].

The TRPA1 role in airway inflammatory disease may be dependent on its expression in non-neuronal cells in human and murine airways (fibroblasts and epithelial cells), where its activation promotes non-neurogenic inflammatory responses [38]. Thus, the TRPA1 channel, with its wide range of expression in neuronal and non-neuronal cells and its activation by several exogenous and endogenous proinflammatory stimuli relevant to airway sensory responses, may be a major regulator in driving several respiratory diseases, including asthma and COPD. Finally, it should be remembered that activation of human TRPA1 in vagal sensory afferents leads to changes in breathing patterns, dyspnea, and cough [86].

## 4. TRPA1 and Rheumatoid Arthritis

Rheumatoid arthritis (RA) is an autoimmune disease that causes chronic inflammation, joint pain, and damage throughout the body, often markedly decreasing the quality of life of patients [87]. Chronic inflammation is a feature of RA [88], a condition characterized by overgrowth of synovial fibroblasts, producing matrix-degrading enzymes and proinflammatory cytokines [89]. N-acylethanolamines reduce the inflammatory mediators originated by synovial cells from RA patients by COX-2 inhibition and concomitant TRPA1 desensitization [90]. The synthetic cannabinoid agonist, WIN55,212-2 mesylate, reduces the release of inflammatory mediators, such as interleukin (IL)-6, IL-8, and matrix metalloproteinase (MMP)-3, in RA synovial fibroblasts via a TRPA1-dependent mechanism [91].

The expression of TRPA1 in peripheral blood leukocytes from patients with RA has been positively correlated with joint pain and disability [92]. Modulation of TRPA1 in RA patients was associated with CD14^+^ cell activation and higher numbers of circulating neutrophils, effects that might contribute to pain and disability in RA patients as the leukocytes populating the joints would amplify the proalgesic process [92]. Stimulation of synovial fibroblasts with tumor necrosis factor alpha (TNF-α) leads to an upregulation of TRPA1 and its sensitization [93], and TRPA1 activation increases Ca^2+^ influx, thus affecting cell viability [93].

In a rodent model of RA of serum transfer, amelioration of inflammation by a sulfide donor (GYY4137) by interaction with TRPA1 has been observed, indicating a therapeutic value of sulfides for RA [94]. Auranofin, which improves arthritis symptoms, including painful joints, and is widely used for the treatment of RA, has been shown to activate the human isoform of TRPA1 [95]. TRPA1 activation mediated by auranofin may be responsible for the adverse effects caused by this drug treatment [95].

Inhibition of TRPA1 in RA synovial fibroblasts by cannabidiol reduces cell viability, proliferation, and cytokine production [96], showing that TRPA1 possesses anti-arthritic activity and may ameliorate arthritis via targeting synovial fibroblasts under inflammatory conditions. There is also evidence that TRPA1 is expressed in human chondrocytes of osteoarthritic patients, and the TRPA1 antagonist significantly downregulated the expression of IL-6 in chondrocytes from wild-type mice and osteoarthritic patients [97]. Together, these findings highlight the role of TRPA1 as a potential mediator and novel drug target in various types of arthritis.

## 5. TRPA1 and Endometriosis

Endometriosis is characterized by the presence of endometrial tissue outside the uterus, which causes chronic inflammatory reactions resulting in scar tissue formation [98]. Endometriosis, associated with debilitating chronic pelvic pain (CPP), is an estrogen-dependent inflammatory disease that affects 5% to 10% of reproductive-age women [99]. Histologically, the endometriotic lesion consists of endometrial-like glands, stroma, and hemosiderin, with blood vessels, nerve fibers, muscle, and immune cells [100]. Women with endometriosis often suffer from cyclic pain, which may be associated with other conditions, such as irritable bowel syndrome and migraine [101,102]. Visceral, mechanical, and generalized hypersensitivity is widespread in women with endometriosis [103]. However, there is no correlation between the extent of disease and reported pain scores. Symptoms associated with the disease can include various pain symptoms, such as chronic pelvic pain and pain during urination and sexual intercourse [104]. The origins of endometriosis are thought to be multifactorial, and various hypotheses have been proposed, including retrograde menstruation and cellular metaplasia. However, other factors can contribute to the growth or persistence of ectopic endometrial tissue. For instance, endometriosis is known to be dependent on estrogen, which facilitates the inflammation, growth, and pain associated with the disease [98,105].

A significant increase in the TRPA1 mRNA content was observed in nociceptive neurons in the peritoneum of women with chronic pelvic pain caused by endometriosis [106]. Increased immunoreactivity of TRPA1, associated with increased mRNA expression in ectopic endometrium of deep infiltrating endometriosis patients, has been detected [107]. In a non-surgical model of endometriosis, the presence of TRPA1 was found in endometriotic lesions infiltrated with macrophages, neutrophils, and mast cells [108]. An increase in TRPA1 mRNA and exaggerated Ca^2+^ responses in dorsal root ganglion (DRG) neurons from mice with endometriosis has been reported [108], suggesting that endometriosis affects protein expression and TRPA1 responsiveness in DRG neurons. The role of TRPA1 as a major sensor [23,24,27] and amplifier of the oxidative burst underlying a variety of inflammatory responses [51,52,54,55,62] supports its implication in endometriosis pain symptoms.

## 6. TRPA1 and Inflammatory Bowel Disease

Inflammatory bowel disease (IBD) encompasses two principal types of inflammatory disorders of the gastrointestinal tract: ulcerative colitis (UC) and Crohn’s disease (CD) [109], which share several common pathophysiologic features (disturbances of the immune system, mucosal barrier function, and gut microbiota) [110] and symptoms (abdominal pain, presence of blood in stool, diarrhea, and weight loss) [111,112]. Although usually not life-threatening, IBD severely affects a patient’s quality of life, especially because of the presence of chronic pain [113,114], which is poorly amenable to pharmacologic treatment [115,116]. The exact pathogenesis of this inflammatory disorder is still unknown.

The contribution of TRPA1 to the pathogenesis of IBD, however, remains unclear, with literature data indicating pro- and anti-inflammatory effects or no influence. It has been shown that TRPA1 contributes to colorectal contraction and visceromotor response after administration of allyl isothiocyanate (AITC) in a colitis model induced by 2,4,6-trinitrobenzenesulfonic acid (TNBS) [117]. The induction of colitis with TNBS leads to oxidative stress generation, which, by TRPA1 activation, results in hypersensitivity to visceromotor response [118]. TRPA1 has also been reported to contribute to visceral pain-like behaviors in dextran sulfate sodium (DSS)-evoked colitis, an effect associated with upregulation of channel expression and responsiveness in DRG nociceptors [119].

In tissue biopsies from patients with active and inactive CD and UC, a significant TRPA1 mRNA upregulation has been found [120,121]. Specifically, TRPA1 has been detected in CD4^+^ T cells infiltrating the colonic tissue samples of both UC and CD patients, where its stimulation controls CD4^+^ T-cell activation and proinflammatory responses, thus suggesting an important contribution of the channel in the pathogenesis of IBD [121]. In an experimental model of TNBS-evoked colitis, the inhibition, or genetic deletion, of TRPA1 reduced the inflammatory process by reducing colonic neuropeptide release (substance P and CGRP) from gut extrinsic sensory neurons [122]. Activation, and the ensuing desensitization of TRPA1 by cannabidivarin (CBDV, a cannabis derivative), seem to have potential intestinal anti-inflammatory effects in mice [123]. CBDV treatment decreased neutrophil infiltration and cytokine production in mice with colonic inflammation induced by dinitrobenzenesulfonic acid (DNBS) [123]. Moreover, CBDV exerts intestinal anti-inflammatory effects in children with active UC [123]. Another study reported opposing results, showing that the absence of TRPA1 worsened the inflammatory process induced by DSS, as its activation exerts protective roles by decreasing the expressions of several proinflammatory neuropeptides, cytokines, and chemokines [120].

Localization of the TRPA1 channel in different cell types of the gastrointestinal tract (extrinsic and enteric neurons, neuroendocrine, and immune cells) and its similar regulation in human and mouse inflammation suggest important channel functions in IBD that, however, need further investigation before the proposal of its potential therapeutic value in these disorders of the gastrointestinal tract.

## 7. TRPA1 and Atherosclerosis

Atherosclerosis (AS) is currently recognized and described as an inflammatory disease [124,125]. AS is characterized by the accumulation of lipids and the formation of atherosclerotic plaques that cause the hardening of the arterial vessel wall and the arterial lumen [126]. The fatty streak of the arterial wall is initially composed almost entirely of monocyte-derived macrophages. The subsequent recruitment of T cells, mast cells, and other inflammatory cells to the intima promote the development of an atheroma [127]. AS plaques can remain stable for years but rapidly become unstable to induce rupture and trigger thrombus formation. Accordingly, in addition to the restriction of the lumen vessel, the presence of atherosclerotic plaques is linked to an increased risk of acute cardiovascular events, such as myocardial infarction and stroke [126].

There is evidence that TRPA1 and cardiovascular diseases have a close relationship, especially in the inflammation underlying these conditions [128]. Adenosine triphosphate (ATP) is a major trigger of AS [129,130] by acting on the P2X7 receptor (P2X7R) to mediate macrophage-dependent inflammation [131]. TRPA1 co-localizes with P2X7R in macrophages, where it contributes to ATP-induced oxidative stress and inflammation. During atherosclerosis, ATP is released in the extracellular matrix, stimulating inflammation and monocyte migration [132]. In particular, ATP and the potent P2X7R agonist 3’-O-(4-Benzoylbenzoyl)-ATP (BzATP) induces macrophage activation, calcium overload, mitochondria injury, IL-1β secretion, and cytotoxicity, all effects that, inhibited by TRPA1 antagonism [133,134,135], indicate a channel role in ATP-induced inflammation in AS [133].

Macrophages orchestrate AS, as their polarization from anti-inflammatory to proinflammatory (M2 and M1, respectively) phenotypes play a key role in AS progression. TRPA1 inhibition would promote macrophage polarization toward an inflammatory phenotype by stimulating M1 and repressing M2 gene expression to modulate the AS plaque progression [136,137]. The relationship that exists between the TRPA1 channel and AS makes the channel activation a potential target to develop new clinical treatments for AS.

## 8. TRPA1 and Inflammatory Skin Diseases

Psoriasis and atopic dermatitis are two of the most common chronic inflammatory skin diseases. Both diseases are caused by a complex interplay between skin-barrier disruption, immune dysregulation, host genetics, and environmental triggers [138,139]. They result in chronic, systemic inflammation with increased circulating lymphocytes, leukocytes, proinflammatory cytokines, and chemokines [140,141]. Atopic dermatitis affects up to 15–20% of children and 1–10% of adults worldwide [142]. Psoriasis affects 2–3% of the global population, corresponding to >125 million individuals [143]. Although they show some differences in their etiology and clinical manifestations, patients with either disease suffer from health-related low quality of life, mainly due to pruritus [144].

TRPA1 can contribute to the pathogenesis of chronic [46] and acute histamine-independent pruritus, such as those evoked in mice by injection of chloroquine [145]. A TRPA1-dependent pathway of itch in atopic dermatitis has been shown in an IL-3-induced mouse model, where a correlation between increased scratching behavior and TRPA1 expression in mast cells, dermal sensory nerve fibers, and cell bodies of DRG neurons has been observed [46]. The pharmacological blockade of the TRPA1 channel has been found to attenuate the scratching [46].

Another mouse model of atopic dermatitis, such as that induced by 2,4-dinitrochlorobenzene (DNCB), implicates TRPA1 activation [146]. DNCB can provoke pathological symptoms, including ear thickness, epidermal hyperplasia, and pruritus, which were attenuated in mice with TRPA1 genetic deletion [147]. Similarly, the induction of atopic dermatitis by topical application of oxazolone in mice induces milder atopic dermatitis symptoms, including pruritus, and lower levels of inflammatory cytokines and T-cell activation via TRPA1 [148]. In fact, oxazolone has been shown to activate TRPA1 directly, inducing the release of mediators of neurogenic inflammation and pruritus, such as 5-hydroxytryptamine (5-HT), SP, and neurokinin A (NKA) [148]. The role of TRPA1 has been highlighted in several pathways involved in chronic allergic itch, including the release of atopic dermatitis-associated cytokines from keratinocytes via a Th2-cell-neuronal mechanism, such as the pruritogenic cytokine IL-31 [149]. A keratinocyte-neuronal axis based on the release of thymic stromal lymphopoietin [150] and periostin [151] has also been proposed.

Recent evidence supports the role of TRPA1 in psoriasis since the application of imiquimod (IMQ) is associated with the expression of TRPA1 in psoriatic lesions observed after drug application [152]. Increased expression of TRPA1 was also observed in psoriatic skin from human subjects where TRPA1 and TRPV1 genes were over-expressed [153]. Paradoxically, pharmacological blockade or genetic deletion of TRPA1 worsen psoriatic dermatitis and nocifensive and itch behavior in mice by increasing inflammatory cytokines, including IL-1β, TNF-α, and IL-22 [154]. In agreement with this data, the activation of TRPA1 by the selective agonist, AITC, reduced the IMQ-induced psoriasiform inflammation, thus suggesting that the presence of TRPA1 mitigates the psoriasis effects [155]. Overall data show that, in psoriasis, TRPA1 exerts an anti-inflammatory role, and its activation, rather than inhibition, could lead to local control of skin inflammation and pruritus observed in both diseases.

## 9. TRPA1 and Neurodegenerative Inflammatory Diseases

Expression of TRPA1 has been reported in various brain areas where it seems to play a modulatory role in neurodegenerative disorders and neuroinflammation, such as multiple sclerosis, Alzheimer’s (AD), and Parkinson’s (PD) diseases [156,157,158,159,160]. The TRPA1 channel has been localized to astrocytes of the corpus callosum [156], in oligodendrocytes of the cerebellum [49], and in cerebral artery endothelium [161,162]. Hippocampal astrocytes also express TRPA1, and TRPA1 immunoreactivity has been found in the cortical neurons of amyloid precursor protein/presenilin1 (APP/PS1) transgenic mice [157]. In humans, TRPA1 has been detected in the cortex, caudate nucleus, putamen, globus pallidus, substantia nigra, cerebellum, amygdala, and hypothalamus [163]. However, few in vivo data are available to corroborate the function of TRPA1 in neurodegenerative inflammatory diseases.

### 9.1. TRPA1 and Alzheimer’s Disease

Alzheimer’s disease (AD) is the most common cause of dementia in the elderly. Pathologically, AD is characterized by protein deposition, extracellularly as amyloid plaques and intracellularly as neurofibrillary tangles [164]. While neurofibrillary tangles are commonly found in several neurodegenerative diseases, amyloid plaques are a specific hallmark of AD. For this reason, the deposition of amyloid has generally been more closely associated with the primary pathogenic mechanism of AD [165]. Amyloid plaques are principally composed of the amyloid beta peptide (Aβ peptide), a 4 kDa polypeptide derived by proteolytic cleavage of the β-amyloid precursor protein [166].

Aggregation of the Aβ peptide induces the release of Ca^2+^ stored in the endoplasmic reticulum (ER), resulting in an overload of cytosolic Ca^2+^. In response to the rise in endogenous Ca^2+^, levels of reduced glutathione (GSH) decrease, leading to intracellular ROS accumulation [167]. In addition, the deposition of Aβ peptide induces microglial activation [168] and the release of a series of cytokines that amplify inflammatory signaling pathways for neuronal damage and death [169]. In the brains of AD transgenic mice, increased levels of TRPA1 have been associated with increased levels of Aβ protein and neuroinflammation [170].

Aβ protein triggers a TRPA1-dependent Ca^2+^ influx, associated with astrocytic activation, with subsequent increase in protein phosphatase 2B activity, nuclear factors of activated T cells (NFAT), and nuclear factor-κB (NFκB). These changes amplify proinflammatory cytokine release [157]. TRPA1 inhibition is sufficient to prevent the insurgence of neuronal hyperactivity. Neuronal hyperactivity seems to be the driving force for initial progressive failures, which leads to the loss of functional dendritic spines and subsequent neuronal dysfunction. The chronic inhibition of the TRPA1 channel has been shown to normalize astrocytic activity, avoid perisynaptic astrocytic process withdrawal, and prevent neuronal dysfunction, thus preserving structural synaptic integrity [171]. These findings support the hypothesis that astrocyte TRPA1 is critical for Alzheimer’s disease progression and suggest TRPA1 antagonists as a potential therapeutic for neuroprotection.

### 9.2. TRPA1 and Parkinson’s Disease

Parkinson’s disease (PD) is the second most common neurodegenerative disorder globally and affects approximately 10 million (0.3%) people (>60 years) worldwide [172]. PD is characterized by a marked loss of dopaminergic neurons in substantia nigra [173]. Although the mechanisms responsible for neuronal degeneration are still unknown, mitochondrial dysfunction, oxidative stress, and glutathione depletion, as well as inflammation, altered calcium homeostasis, aggregation, excitotoxicity [174], and activation of microglia mediated by glucocorticoid receptors [175,176], seem to have a role. Considering that oxidative stress and changes in Ca^2+^ homeostasis are involved in PD, TRPA1 has been reported to mediate some of the mechanisms that lead to disease progression [177]. Acrolein, a well-known TRPA1 agonist [33], seems to have a role in PD. Acrolein content was elevated in a rat model of PD evoked by 6-hydroxydopamine (6-OHDA) [178], and the acrolein scavenger, dimercaprol, produced a neuroprotective effect [179]. Dimercaprol-mediated suppression of acrolein was associated with a significant reduction in neuronal loss in both the striatum and substantia nigra in 6-OHDA rats [180]. Additional studies corroborate this finding by showing that injecting acrolein into a rat brain reproduced PD-like symptoms and pathological signs mirroring those seen in 6-OHDA-injected rats. Lowering acrolein levels via another scavenger (hydralazine) mitigates PD pathologies and motor deficits [181]. There is evidence that other TRP channels, such as TRPM2 and TRPM7 [182,183], are activated during PD progression [184]. Their activation triggers multiple events, such as increased intracellular Ca^2+^ ions, neuronal inflammation, mitochondrial dysfunction, and DNA damage, leading to the activation of the apoptotic pathway and neuronal cell death.

### 9.3. TRPA1 and Multiple Sclerosis

Multiple sclerosis (MS) is a chronic inflammatory autoimmune disease affecting patients’ physical/cognitive status through neurodegeneration and demyelination in the central nervous system (CNS). With a prevalence of 1 in 3000 people, 2.8 million individuals were estimated to live with multiple sclerosis worldwide in 2020 [185]. MS is characterized by several debilitating symptoms, including muscle weakness, blurred vision, vertigo, fatigue, balance problems, and various types of pain [186,187]. Several studies have reported that primary headaches, such as migraine and tension-type headaches, are more frequent in MS patients than in the general population [188,189,190].

Genetic and pathological studies indicate that T and B cells are implicated in the pathogenesis of MS [191]. Alterations in cytokine production, T and B cell co-stimulation likely contribute to the disruption of the blood–brain barrier, thus allowing lymphocyte infiltration into the CNS and causing multifocal inflammation and demyelination, oligodendrocyte loss, reactive gliosis, and the production of the cytotoxic agents, ROS and RNS, to sustain neuroaxonal degeneration [192]. Astrocyte activation, macrophage infiltration, and mitochondrial dysfunction at sites of MS lesions generate oxidative stress, which has been considered to play a major role in the mechanism of demyelination. Notably, in homogenates of MS white and gray matter, demyelination has been associated with increased myeloperoxidase activity [193]. Widespread oxidative damage in demyelinating MS plaques is documented by the expression of markers of oxidative damage, such as 4-HNE [194]. 4-HNE accumulates in both phagocytic macrophages and large hypertrophic astrocytes of active demyelinating MS lesions [195].

In the CNS, TRPA1 expression has been shown in astrocytes [156] as well as oligodendrocytes [49]. In a model of MS induced by cuprizone, TRPA1 deficiency significantly attenuated cuprizone-induced demyelination by reducing the apoptosis of mature oligodendrocytes [156]. Specifically, the activation of TRPA1 in astrocytes by enhancing the intracellular Ca^2+^ concentration seems to influence the pro-apoptotic pathways in oligodendrocytes. Thus, a modulatory effect on apoptosis by an interaction between oligodendrocytes and astrocytes can occur. It has also been reported that TRPA1 activation can profoundly modulate physiological astrocyte functions [196] and that astrocytes (by releasing biologically active molecules) have a direct effect on oligodendrocyte death or survival [197,198,199].

The beneficial effect of TRPA1 inhibition on neuroglial cell activation and demyelination has also been shown by a recent computational study, which, by molecular docking techniques, potential TRPA1 selective inhibitors, including desvenlafaxine, paliperidone, and febuxostat (which possess a suitable blood–brain barrier (BBB) permeability) have been reported as the most promising repurposable agents for treating MS [200]. On the other hand, additional in vitro and in vivo tests are necessary to confirm the biological action of these novel molecules on demyelination. In a model of relapsing-remitting experimental autoimmune encephalomyelitis, TRPA1, presumably activated by endogenous agonists, was shown to be involved in the development of periorbital mechanical allodynia, a hallmark of MS headache [201]. In addition, a selective TRPA1 antagonist diminished the depression- and anxiety-like behaviors in a mouse model of progressive MS [202]. Thus, inhibition of TRPA1 receptors might attenuate neuronal degeneration by limiting demyelination and could be a promising therapeutic target to limit the development of the physical/cognitive impairment and painful conditions associated with MS.

## 10. Conclusions

TRPA1, expressed by primary sensory neurons and inflammatory, and immune cells, detects and is activated by a series of endogenous proinflammatory molecules. These molecules are implicated in modulating intracellular processes in inflammatory diseases. A number of these molecules are byproducts of oxidative, nitrative, and carbonylic stress, which non-specifically affect and damage nucleic acids, lipids, and proteins. However, these same compounds have recently been identified as signaling molecules, which, via TRPA1 targeting, affect inflammatory and immune responses (Table 1). Thus, given its expression in various types of tissues and cells, and its ability to activate a variety of biological responses, TRPA1 is considered a novel and attractive therapeutic target for the treatment of human inflammatory diseases (Table 1).

## Figures and Tables

**Table 1:** TRPA1 in inflammatory diseases.

Inflammatory Diseases	TRPA1 Distribution	TRPA1 Activation-Dependent Effect	References
Asthma and COPD	Vagal sensory neurons, lung fibroblasts, and epithelial cells	Vagal nerve activation, cough, bronchoconstriction, airway neurogenic inflammation	[40,74,75,76,77,78,79,80,84]
Rheumatoid arthritis	Peripheral blood leukocytes, synovial fibroblasts	Increase in cell viability and proliferation, release of inflammatory mediators	[94,95,98]
Endometriosis	Peritoneum nociceptive neurons, stromal and epithelial cells of ectopic endometrium	Increase in Ca^2+^ responses and oxidative stress, increase in pain hypersensitivity	[108,109,110]
Inflammatory bowel disease	Extrinsic and enteric neurons, neuroendocrine cells, colonic tissue CD4+ T cells	Increase in pain hypersensitivity, release of proinflammatory neuropeptides, cytokines, and chemokines	[120,121,122,123,124]
Atherosclerosis	Macrophages in atherosclerosis plaque	M1 macrophages polarization, calcium overload, mitochondria injury, increase in IL-1β secretion, and oxidative stress	[135,136,137,138,139]
Psoriasis and atopic dermatitis	Mast cells, dermal sensory nerve fibers, keratinocytes, melanocytes	Release of inflammatory cytokines, pruritus	[48,149,150,151,156,157]
Alzheimer’s and Parkinson’s diseases	Astrocytes, oligodendrcocytes, cerebral artery endothelium, dopaminergic neurons	Increase in Ca^2+^ response, astrocyte activation, increased levels of Aβ peptide and neuroinflammation, increase in oxidative stress	[51,158,159,163,164,165,173,179,180,181,182,183]
Multiple Sclerosis	Astrocytes, oligodendrocytes	Modulation of pro-apoptotic pathways, increased Ca^2+^ influx, neuroglial activation, periorbital allodynia	[51,158,198,199,200,201,202]
